# Supplementary material for: Boosting the Photocatalytic Ability of TiO2 Nanosheet Arrays for MicroRNA-155 Photoelectrochemical Biosensing by Titanium Carbide MXene Quantum Dots
Source: Nanomaterials (Basel). 2022 Oct 11;12(20):3557. doi: 10.3390/nano12203557 (PMC9611374; doi:10.3390/nano12203557)
Supplement: Supplementary file 1 [file nanomaterials-12-03557-s001.zip › nanomaterials-1939728-supplementary.pdf]

*Supporting Information*

# Boosting the Photocatalytic Ability of $TiO_2$ Nanosheet Arrays for MicroRNA-155 Photoelectrochemical Biosensing by Titanium Carbide MXene Quantum Dots

**Bingdong Yan [1], Zike Cheng [1], Caiyan Lai [1], Bin Qiao [2], Run Yuan [1], Chide Zhang [1], Hua Pei [2], Jinchun Tu [1,*] and Qiang Wu [2,*]**

[1] State Key Laboratory of Marine Resource Utilization in South China Sea, School of Materials Science and Engineering, Hainan University, Haikou 570228, China

[2] Department of Clinical Laboratory of the Second Affiliated Hospital, School of Tropical Medicine, Key Laboratory of Emergency and Trauma of Ministry of Education, Research Unit of Island Emergency Medicine, Chinese Academy of Medical Sciences (No. 2019RU013), Hainan Medical University, Haikou 571199, China

* Correspondence: tujinchun@hainanu.edu.cn (J.T.); wuqiang001001@aliyun.com (Q.W.)

## Materials and reagents

Fluorine-doped tin oxide (FTO; 1 cm × 2 cm) thin-film substrate was obtained from Opivit New Energy Technology Co., Ltd. (China). Hydrochloric acid, tetrabutyl titanate, titanium trichloride, ethanol, tetraethyl orthosilicate, and 3-aminopropyl triethoxysilane were obtained from Sinopharm Chemical Reagent Co., Ltd. (China). S9.6 antinody (anti-DNA:RNA antibody) was supplied by KeraFAST (USA) and diluted in phosphate-buffered saline (0.1 M $Na_2HPO_4$, 0.15 M NaCl, and 0.05% (w/v) $NaN_3$; pH 7.4). Au NPs were supplied by Nanjing XFNANO material Technology Co., Ltd. (China). Ascorbic acid-2-phosphate (AAP), ascorbic acid (AA) and Mercaptohexanol (MCH) was provided by Solarbio (Beijing, China). IgG-ALP was purchased from Sangon Biotech (Shanghai, China).

The buffer solutions used in this work were as follows:

(I) probe-immobilization buffer, 10 mM Tris–HCl, 1.0 mM EDTA, 1.0 M NaCl, and 1.0 mM TCEP (pH 7.4);

(II) microRNA (miRNA) hybridization buffer, 1 × SSC (0.15 M sodium chloride and 15 mM sodium citrate);

(III) washing buffer, 10 mM Tris–HCl containing 50 mM KCl; and

(IV)detection solution, 10 mM Tris–HCl (pH 9.8) containing 50 mM KCl, 0.1 M AAP, and 0.1 mM $Mg(NO_3)_2$.

All solutions and redistilled deionized water used were treated with DEPC and autoclaved to protect from RNase degradation.

The oligonucleotide sequences, fabricated by Sangon Biotech Co., Ltd. (China), were as follows (Table S1).

**Table S1.** Specific oligonucleotide sequence.

| Oligonucleotide | Sequences (from 5′ to 3′) |
|---|---|
| Probe DNA | 5′-SH-$(CH_2)_6$-ACC CCT ATC ACG ATT AGC ATT AA-3′ |
| microRNA-155 | 5′-UUA AUG CUA AUC GUG AUA GGG GU-3′ |
| microRNA-21 | 5′-UAG CUU AUC AGA CUG AUG UUG A-3′ |
| microRNA-122 | 5′-UGG AGU GUG ACA AUG GUG UUU G-3′ |
| microRNA-141 | 5′-UAA CAC UGU CUG GUA AAG AUG G-3′ |

## Material Characterization

The morphology and structure information of the products were examined with a Sirion 200 field-emission scanning electron microscopy (FE-SEM) system and a transmission electron microscopy (TEM) system (FEI Tecnai G230, Hillsboro, OR, USA), respectively. In a typical FE-SEM measurement, a small piece of sample film was adhered onto a copper stub by using double-sided carbon tape. The specimens for TEM imaging were prepared by suspending solid samples in acetone. About 1–2 mg of white sample detached from the FTO substrate was added to 5 mL of acetone in a small glass vial, followed by sonication for 30 min. A few drops of the sonicated suspension were added onto a carbon-coated 200 mesh copper grid, which was dried under ambient conditions before imaging. The XRD patterns of the as-prepared films were recorded on a Philip X'pert X-ray diffractometer (PANalytical, Almelo, the Netherlands) (CuKa irradiation, $\lambda$= 0.15418 nm) from 10° to 80° at a scanning speed of 2.4°/min. X-ray photoelectron spectra were obtained from Thermo Scientific ESCALAB250 by using an Al target. Valance-band spectra were measured with a monochromatic He I light source (21.22 eV) and a VG Scienta R4000 analyzer. A sample bias of −5.0 V was applied to observe the secondary electron cutoff. The work function ($\phi$) can be determined by the difference between the photon energy and the binding energy of the secondary cutoff edge. UV–vis absorption spectra were obtained on a UV–vis absorption spectrophotometer (UV–vis, Thermo Fisher Evolution 220) within the test range of 300–600 nm. PL and TRPL measurements were made on an FL3-P-TCSPC time-resolved fluorescence spectrometer (Horiba Jobin Yvon, Longjumeau, France), the excitation wavelength was 350 nm, the test range was 360–500 nm, and the emission wavelength was 350 nm. PEC measurements were performed with a homemade PEC system. A 395 nm LED lamp photosource served as the irradiation source (Beijing Ceaulight Technology Co., Ltd., China). Photocurrent was recorded on a CHI660A electrochemical workstation (Austin, TX, USA). A conventional three-electrode electrochemical system was used with Ag/AgCl and Pt foil (1 cm × 1 cm) as counter and reference electrodes, respectively. The electrode was tested under off-on-off (10 s–10 s–10 s) switching light at 0.0 V *vs.* Ag/AgCl. Electrochemical impedance spectroscopy was performed in 5.0 mM $Fe(CN)_6^{3-/4-}$ (1:1) solution containing 0.1 M KCl at an open circuit potential over a frequency range $10^{-1}$–$10^5$ Hz

## Characterization of PEC electrode

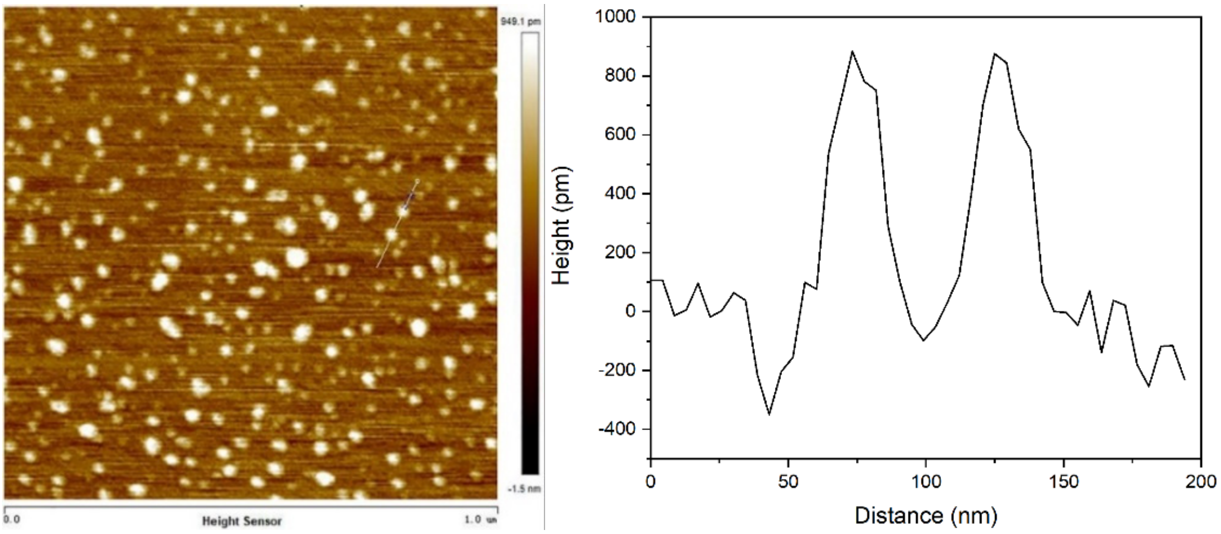

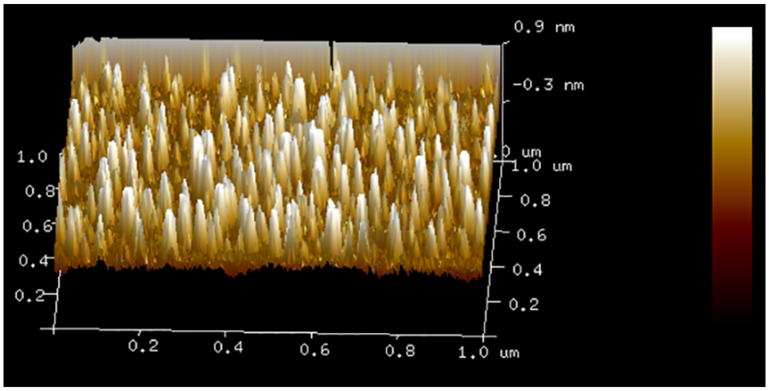


**Figure S1.** Atomic force microscopy (AFM) image of $Ti_3C_2T_x$ QDs.

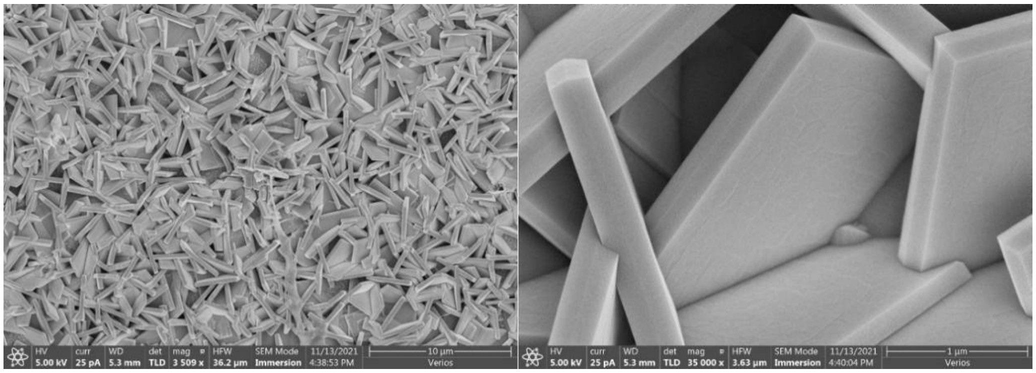

**Figure S2.** FESEM of (001) $TiO_2$ NSs.

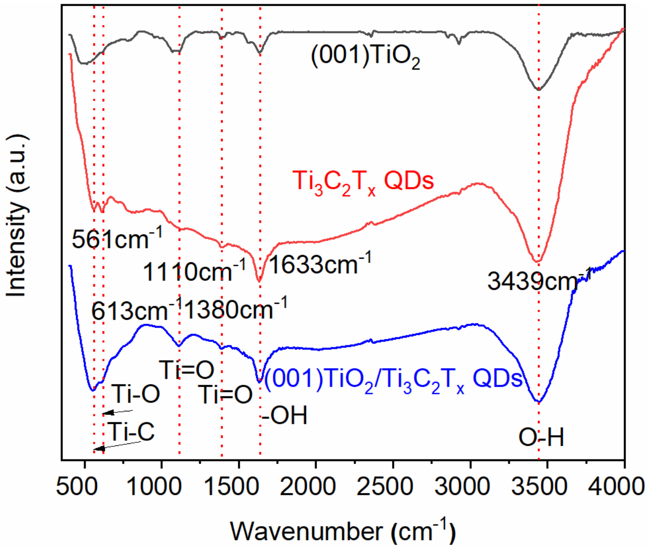


**Figure S3.** FTIR spectra of $TiO_2$ NSs, $Ti_3C_2T_x$ QDs, and $Ti_3C_2T_x$ QDs/(001) $TiO_2$ composite.

**PEC performance characterization of $Ti_3C_2T_x$ QDs/(001) $TiO_2$/FTO electrode**

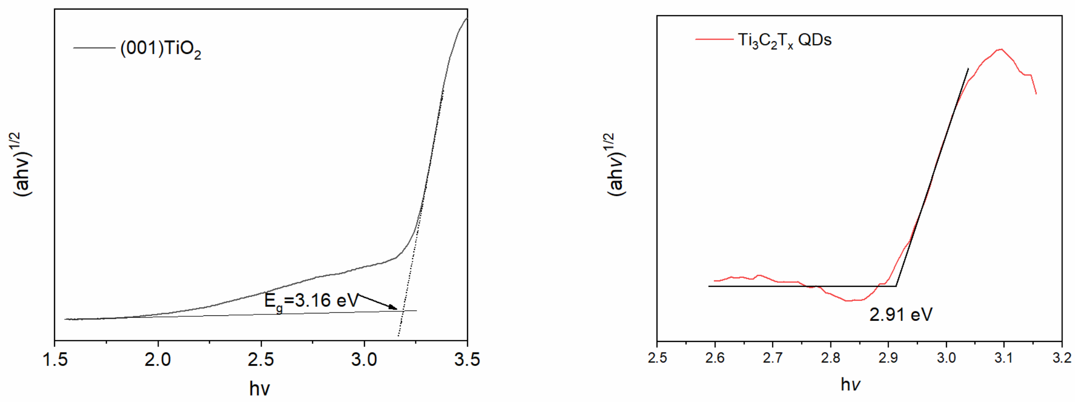


**Figure S4.** UV−vis DRS of (001) $TiO_2$ and $Ti_3C_2T_x$ QDs,.
